# Association between vitamin D receptor gene polymorphisms and genetic susceptibility to benign prostatic hyperplasia: A systematic review and meta-analysis

**DOI:** 10.1097/MD.0000000000037361

**Published:** 2024-03-01

**Authors:** Li Ruan

**Affiliations:** a Department of Urology, Guangzhou Red Cross Hospital (Guangzhou Red Cross Hospital of Jinan University).

**Keywords:** benign prostatic hyperplasia, BPH, gene polymorphisms, vitamin D receptor gene

## Abstract

**Background:**

Benign prostatic hyperplasia (BPH) is one of the global public health challenges due to the complexity of its mechanisms of occurrence. Many studies have suggested that vitamin D receptor gene polymorphisms are associated with BPH susceptibility. Still, their conflicting findings need to be analyzed in aggregate to gain a better understanding.

**Methods:**

We identified 10 trials involving 1539 BPH cases and 1915 controls through a systematic search of Embase using, data obtained from the Web of Science, PubMed, and China Knowledge Network databases as of December 31, 2021. A meta-analysis was performed to investigate the association between 4 constant polymorphisms of this associated vitamin D receptor gene (Fok-1, Bsm-1, Taq-1, and Apa-1) and BPH risk.

**Results:**

In the overall population analysis, a significant positive association with BPH risk was found only in the Taq-1 variant (*P* < .001). Of these, the pure-hybrid model (95% confidence interval [CI] = 1.384–3.196), the heterozygous model (95% CI = 1.207–2.021), the dominant model (95% CI = 1.312–2.133) and the allelic inheritance model (95% CI = 1.205–1.730) showed low heterogeneity. In subtype analyses, Bsm-1 variants showed a significant association with BPH risk for both the recessive (95% CI = 0.100–0.943, *P* = .039) and over-dominant (95% CI = 1.553–3.100, *P* = 0) models in the Caucasian population, and for the recessive (95% CI = 1.242–3.283, *P* = .039) and over-dominant (95% CI = 0.281–0.680, *P* = 0) models in the Asian population. In addition, a high degree of heterogeneity was found in the subgroup analysis of the association between Fok-1 variants and BPH risk.

**Conclusion:**

Overall, there is an association between vitamin D receptor polymorphisms and BPH risk. Identification of BPH susceptibility by vitamin D receptor gene polymorphisms has potential.

## 1. Introduction

Benign prostatic hyperplasia (BPH), a common benign neoplastic disease in aging male, has highly complex mechanisms and has become a significant worldwide public health issue.^[1]^ Although many studies have been conducted in the past decades, the etiology of BPH cannot be completely explained. Moreover, as the population grows and ages, BPH brings with it a greater burden of health care and financial expense.^[2]^ Lower urinary tract symptoms due to clinical BPH include nocturia, urgency, and frequency, which can obstruct bladder outlet, resulting in declining quality of life.^[3,4]^ Some experiments suggested that the risk of BPH might be associated with many factors, such as genetics, hormones, age, smoking, inflammation, and diet, among others.^[2,5,6]^

Rapid advances in molecular biology techniques have led to the introduction of genetic polymorphisms, which have brought enormous benefits to BPH diagnosis. Many studies have focused on the relationship between vitamin D receptor (VDR) and the risk of BPH.^[7,8]^ This gene has been shown to have essential functions in many diseases; such as cancer, cardiovascular disease, and tuberculosis.^[9]^ First, Vit D is synthesized in the skin and mediates many actions in many tissues in the body.^[10]^ It is metabolized to 1,25-dihydroxyvitamin D, which can regulate calcium and phosphate metabolism. Then some gene expressions are regulated after 1,25-dihydroxyvitamin D combining with VDR.^[11]^ Previous studies also found that low levels of vitamin D could be a risk of BPH.^[8]^ The VDR gene is a member of the steroid hormone receptor superfamily and is found on chromosome 12, which has the 4 most common polymorphic loci: Fok-1, Bsm-1, Taq-1, and Apa-1.^[12]^ It activates the vitamin D and forms a heterodimer complex that binds to the vitamin D response element. This product leads to the transcriptional down-regulation of many genes and is thus involved in the development and progression of disease.^[8,13,14]^

**Figure 1.**
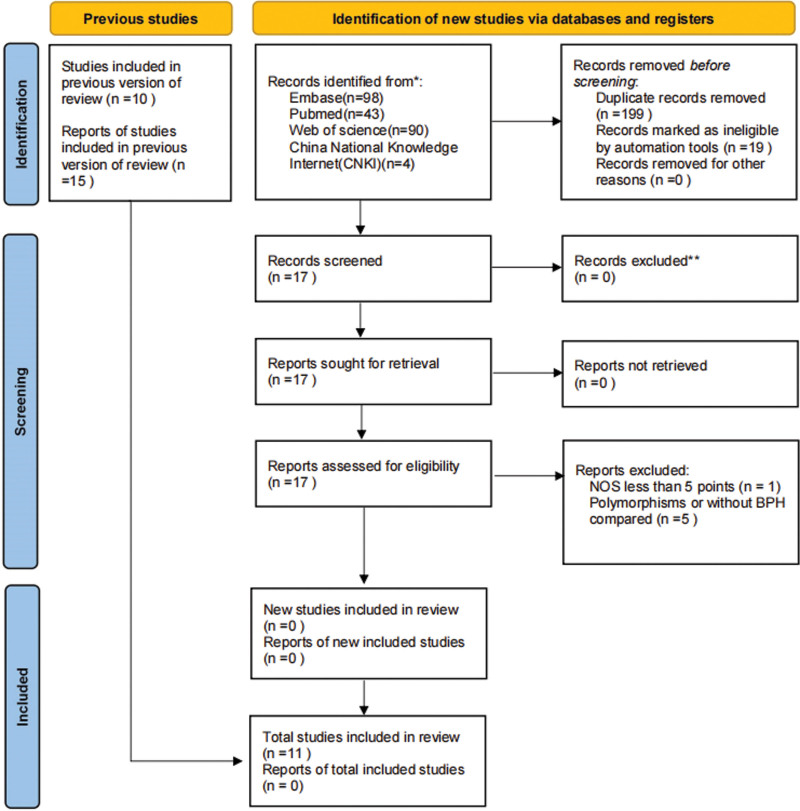
Flowchart of study selection based on the inclusion and exclusion criteria.

Although genome-wide correlation studies have estimated the correlations between VDR polymorphisms and BPH in multiple populations, many findings remain conflicting.^[7,15]^ El-Ezzi et al^[16]^ and Zhang et al^[17]^ presented similar results, suggesting that VDR polymorphisms may play a key role in predicting BPH in Lebanese and Chinese men. However, Bousemaa et al^[15]^ came at the opposite conclusion. Zeng et al,^[9]^ who conducted a meta-analysis on the association of VDR polymorphisms with the risk of BPH 8 years ago, did not reach definitive conclusions and stated that there were significant limitations. This required an updated and comprehensive pooled analysis of these studies. Therefore, a meta-analysis of 10 relative papers on VDR polymorphisms associated with BPH was conducted with the aim of identifying more accurate and reliable results (Fig. 1).^[7,8,13,15–21]^

## 2. Method

### 2.1. Systematic retrieval strategy

We performed a comprehensive search for eligible studies on public databases of Embase (https://www.embase.com/), Web of science (https://www.webofscience.com/wos/woscc/basic-search), PubMed (https://pubmed.ncbi.nlm.nih.gov/), and China National Knowledge Internet (https://www.cnki.net/) from their initial dates to December 2022. The search terms in PubMed were “vitamin D receptor gene or VDR or 1-25 dihydroxycholecalciferol receptor gene,” “gene polymorphisms” and “BPH or benign prostatic hyperplasia.” According to the features of each electronic database, we also manually searched for all possible articles.

### 2.2. Inclusion criteria

The trials chosen must meet the following standards: case-control or cohort study; estimating the relationship between VDR polymorphisms and the BPH risk; offering genotype or allele frequency of VDR polymorphisms that can be assessed odds ratio (OR) and confidence interval (95% CI); literature was written in English and Chinese; the total score must be more than 5 points by using the Newcastle Ottawa Scale.

### 2.3. Data extraction

One author collected sufficient data from eligible researches, the details included the author’s name, publication county, year, race, study design, genotyping methods, source of controls, the number of attendees, Hardy–Weinberg equilibrium in cases and controls, the distributions of genotypes and alleles (Table 1).

**Table 1:** Characteristics of included studies.

Study	Country	Ethnicity	Year	Study design	Genotype method	Source of control	Case-control	HWE	NOS
Chaimuangraj et al^[7]^	Thailand	Asian	2006	Case-control	PCR	HB	44/30	Y	6
Manchanda et al^[8]^	India	Caucasian	2010	Case-control	PCR-RFLP	HB	160/160	N	6
Huang et al^[13]^	China	Asian	2006	Case-control	PCR-RFLP	HB	189/502	Y	5
Bousemaa et al^[15]^	Netherland	Caucasian	2000	Case-control	PCR-RFLP	HB	93/56	Y	6
Hamasaki et al^[22]^	Japan	Asian	2002	Case-control	PCR	HB	83/90	Y	6
El-Ezzi et al^[16]^	Lebanon	Caucasian	2014	Case-control	PCR-RFLP	HB	68/79	Y	6
Zhang et al^[17]^	China	Asian	2017	Case-control	PCR-RFLP	HB	452/501	Y	6
Ruan et al^[18]^	China	Asian	2015	Cohort	PCR-RFLP	HB	200/200	Y	5
Habuchi et al^[20]^	Japan	Asia	2000	Case-control	PCR-RFLP	PB	209/128	Y	6
Nunes et al^[21]^	America	Caucasian	2016	Case-control	PCR-RFLP	BP	41/169	Y	6

HB = hospital-based, HWE = Hardy–Weinberg equilibrium, N = non-HWE, NOS = Newcastle–Ottawa Scale, Y = HWE.

### 2.4. Statistical analysis

The *P* values of HWE in each study were recomputed by using the Chi-square test in SPSS software version 13.0 (SPSS Inc., Chicago, IL). The pooled OR and its 95% CI in 5 genetic models were calculated to evaluate the relationship between VDR gene polymorphisms and BPH risk. *Z* test identified the statistical significance of OR. homozygote model (WW vs ww), heterozygous model (Ww vs ww), dominant model (WW vs Ww/ww), over-dominant model (Ww vs WW/ww), recessive model (WW/Ww vs ww), and allele genetic model (W vs w allele) were conducted to estimate in our analysis. In addition, we used Cochran Chi-square-based *Q* statistic and the inconsistency index (*I*^2^) to evaluate heterogeneity, the random-effects model was chosen appropriately because of *P* < 5% or *I*^2^ > 50%. Otherwise, a fixed-effects model was selected. Subgroup analysis based on the characteristics of ethnicities as well as sensitivity analysis were implemented in each outcome. Furthermore, we used Begg and Egger test to quantitatively estimate publication bias, *P* < 0.05 were considered publication bias. All data analysis were undertaken by using Stata version 12 (StataCorp LP, College Station, TX).

### 2.5. Data availability

All data generated or analyzed during this study are included in this published article.

## 3. Results

### 3.1. Associations between VDR gene polymorphisms and the BPH risk

A combined analysis with 5 studies for the Apa-1 variant is shown in Table 2. In detail, no heterogeneity for Apa-1 except allelic model (*I*^2^ = 62.6%) and null significant association between Apa-1 variant and the risk of BPH were observed. Fixed effect model was used for subgroup analysis stratified by ethnicity, in which we found significant differences in allele genetic model in Caucasian population (W vs w allele: OR = 1.561, 95% CI = 1.096–2.223, *P* = .013).

**Table 2:** Results of the association between Apa-1 polymorphism and BPH risk in different ethnicities.

Comparison	Studies	Overall effect			Heterogeneity		Public bias	
		OR (95% CI)	Z-score	*P* value	*I*^2^ (%)	*P* value	Begg test	Egger test
Whole								
WW vs ww	5	1.078 (0.731–1.591)	0.38	.704	0	.632	0.806	0.998
WW vs Ww	5	0.962 (0.753–1.229)	0.31	.756	30.7	.217	0.806	0.172
WW vs Ww/ww	5	0.953 (0.755–1.202)	0.41	.684	34.7	.19	0.806	0.246
ww vs WW/Ww	5	1.003 (0.753–1.335)	0.02	.986	0	.749	0.462	0.093
Ww vs WW/ww	5	1.039 (0.842–1.282)	0.36	.721	18.3	.298	0.086	0.038
W vs w	5	1.123 (0.830–1.519)	0.75	.454	62.6	.03	0.806	0.246
Caucasian								
WW vs ww	2	1.294 (0.630–2.658)	0.7	.483	0	.714		
WW vs Ww	2	1.669 (0.908–3.068)	1.65	.099	0	.483		
WW vs Ww/ww	2	1.566 (0.885–2.770)	1.54	.124	0	.493		
ww vs WW/Ww	2	1.041 (0.608–1.782)	0.15	.884	0	.901		
Ww vs WW/ww	2	0.718 (0.448–1.153)	1.37	.17	0	.389		
W vs w	2	1.561 (1.096–2.223)	2.47	.013	0	.38		
Asian								
WW vs ww	3	0.999 (0.629–1.586)	0	.997	3.7	.354	1	0.466
WW vs Ww	3	0.864 (0.661–1.130)	1.07	.286	0	.477	1	0.466
WW vs Ww/ww	3	0.865 (0.671–1.115)	1.12	.261	4.5	.351	1	0.626
ww vs WW/Ww	3	0.988 (0.704–1.386)	0.07	.943	0	.389	0.296	0.117
Ww vs WW/ww	3	1.140 (0.900–1.442)	1.09	.277	0	.533	0.296	0.035
W vs w	3	0.942 (0.712–1.247)	0.42	.677	43.1	.172	1	0.999

Homozygote model, WW vs ww; heterozygous model, WW vs Ww; dominant model, WW vs Ww/ww; recessive model, ww vs WW/Ww; over-dominant model, Ww vs WW/ww; allele genetic model, W vs W.

CI = confidence interval, OR = odds ratio.

Table 3 shows the fundamental relation between Bsm-I polymorphism and BPH. Fixed effect model was chosen for data analysis when *I*^2^ < 50%. Pooled results were not associated with BPH significantly under all comparison models, but contrary to recessive model (WW/Ww vs ww: OR = 0.307, 95% CI = 0.100–0.943, *P* = .039), over-dominant model (Ww vs WW/ww: OR = 2.194, 95% CI = 1.553–3.100, *P* = 0) in the subgroup of Caucasian population as well as recessive model (WW/Ww vs ww: OR = 2.019, 95% CI = 1.242–3.283, *P* = .039), over-dominant model (Ww vs WW/ww: OR = 0.437, 95% CI = 0.281–0.680, *P* = 0) in the subgroup of Asian population.

**Table 3:** Results of the association between Bsm-1 polymorphism and BPH risk in different ethnicities.

Comparison	Studies	Overall effect			Heterogeneity		Public bias	
		OR (95% CI)	Z-score	*P* value	*I*^2^ (%)	*P* value	Begg test	Egger test
Whole								
WW vs ww	5	1.774 (0.626–5.028)	1.08	.281	77.4	.001	0.462	0.77
WW vs Ww	5	0.788 (0.546–1.138)	1.27	.204	45.5	.119	0.462	0.302
WW vs Ww/ww	5	0.860 (0.605–1.222)	0.84	.4	11.6	.34	0.462	0.325
ww vs WW/Ww	5	0.599 (0.199–1.803)	0.91	.362	91.3	0	0.462	0.194
Ww vs WW/ww	5	1.371 (0.588–3.200)	0.73	.465	88.8	0	0.806	0.706
W vs w	5	1.068 (0.664–1.717)	0.27	.787	81.40%	0	0.806	0.664
Caucasian								
WW vs ww	3	2.717 (0.543–13.595)	1.22	.224	86.8	.001	0.296	0.569
WW vs Ww	3	0.678 (0.454–1.013)	1.9	.058	44.5	.165	1	0.67
WW vs Ww/ww	3	0.769 (0.524–1.126)	1.35	.177	0.2	.367	1	0.576
ww vs WW/Ww	3	0.307 (0.100–0.943)	2.06	.039	84.3	.002	1	0.241
Ww vs WW/ww	3	2.194 (1.553–3.100)	4.45	0	7.3	.34	1	0.412
W vs w	3	1.372 (0.798–2.359)	1.14	.252	79.9	.007	1	0.439
Asian								
WW vs ww	2	0.881 (0.354–2.190)	0.27	.784	0	.994		
WW vs Ww	2	1.841 (0.693–4.894)	1.22	.221	0	.54		
WW vs Ww/ww	2	1.669 (0.647–4.303)	1.06	.289	0	.543		
ww vs WW/Ww	2	2.019 (1.242–3.283)	2.84	.005	9.9	.292		
Ww vs WW/ww	2	0.437 (0.281–0.680)	3.67	0	0	.343		
W vs w	2	0.618 (0.438–0.872)	2.74	.006	0	.425		

Homozygote model, WW vs ww; heterozygous model, WW vs Ww; dominant model, WW vs Ww/ww; recessive model, ww vs WW/Ww; over-dominant model, Ww vs WW/ww; allele genetic model, W vs W.

CI = confidence interval, OR = odds ratio.

**Figure 2.**
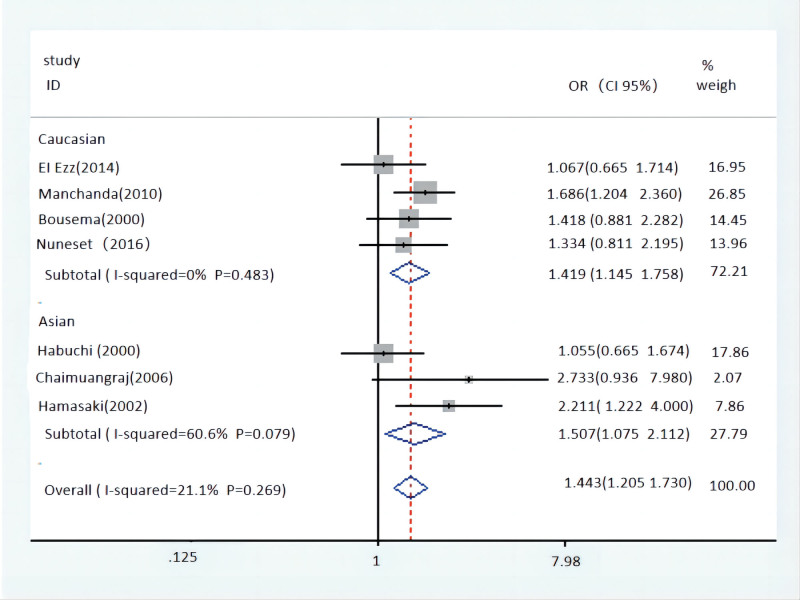
Forest plot of Tap-1 polymorphism and overall population with homozygote model (WW vs ww). CI = confidence interval, OR = odds ratio.

**Figure 3.**
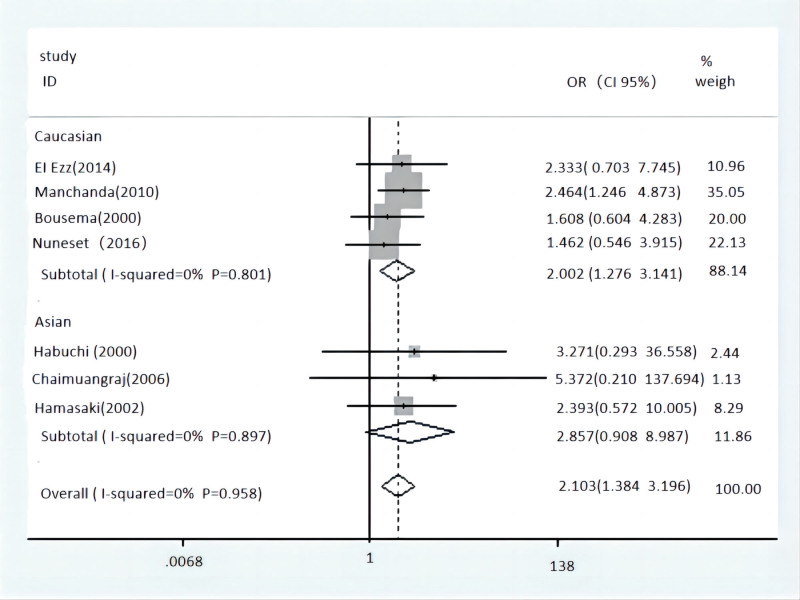
Forest plot of Tap-1 polymorphism and overall population with the allelic model (W vs w). CI = confidence interval, OR = odds ratio.

Outcomes of pooled analysis on the relevance between Taq-1 polymorphism and the BPH risk are displayed in Table 4. The result showed Taq-1 polymorphism could increase the risk of BPH in the multiple populations in the case of low heterogeneity, comparing of homozygote model (WW vs ww: OR = 2.194, 95% CI = 1.384–3.196, *P* = 0) (Fig. 2), heterozygous model (WW vs Ww: OR = 1.562, 95% CI = 1.207–2.021, *P* = .001), dominant model (WW vs Ww/ww: OR = 1.673, 95% CI = 1.312–2.133, *P* = 0), and allele genetic model (W vs w allele: OR = 1.443, 95% CI = 1.205–1.730, *P* = 0) (Fig. 3). However, recessive model (WW/Ww vs ww: OR = 0.558, 95% CI = 0.392–0.795, *P* = .001) got a contrary result. Subsequently, Taq-1 variant confirmed similar effect in Caucasian population, such as homozygote model (WW vs ww: OR = 2.002, 95% CI = 1.276–3.141, *P* = .003), heterozygous model (WW vs Ww: OR = 1.674, 95% CI = 1.188–2.360, *P* = .003), dominant model (WW vs Ww/ww: OR = 1.800, 95% CI = 1.310–2.475, *P* = 0), allele genetic model (W vs w allele: OR = 1.419, 95% CI = 1.145–1.758, *P* = .001) and recessive model (WW/Ww vs ww: OR = 0.577, 95% CI = 0.398–0.837, *P* = .004). In Asian population, the result showed significant association in dominant model (WW vs Ww/ww: OR = 1.507, 95% CI = 1.033–2.199, *P* = .033) as well as allele genetic model (W vs w allele: OR = 1.507, 95% CI = 1.075–2.112, *P* = .017). When analyzing in relevance between Taq-1 polymorphism and the volume of prostate, a result included 2 trials showed no association in recessive model (WW/Ww vs ww: OR = 1.284, 95% CI = 0.489–0.837, *P* = 3.376).

**Table 4:** Results of the association between Tap-1 polymorphism and BPH risk in different ethnicities.

Comparison	Studies	Overall effect			Heterogeneity		Public bias	
		OR (95% CI)	Z-score	*P* value	*I*^2^(%)	*P* value	Begg test	Egger test
Whole								
WW vs ww	7	2.103 (1.384–3.196)	3.48	0	0	.958	0.368	0.413
WW vs Ww	7	1.562 (1.207–2.021)	3.39	.001	27.8	.216	1	0.461
WW vs Ww/ww	7	1.673 (1.312–2.133)	4.15	0	8.6	.363	0.548	0.374
ww vs WW/Ww	7	0.558 (0.392–0.795)	3.24	.001	0	.451	0.764	0.868
Ww vs WW/ww	7	0.760 (0.488–1.183)	1.22	.224	68.2	.004	0.23	0.46
W vs w	7	1.443 (1.205–1.730)	3.98	0	21.1	.269	0.23	0.522
Caucasian								
WW vs ww	4	2.002 (1.276–3.141)	3.02	.003	0	.801	0.734	0.482
WW vs Ww	4	1.674 (1.188–2.360)	2.94	.003	0	.466	1	0.804
WW vs Ww/ww	4	1.800 (1.310–2.475)	3.62	0	0	.929	1	0.837
ww vs WW/Ww	4	0.577 (0.398–0.837)	2.9	.004	41.8	.161	0.734	0.065
Ww vs WW/ww	4	0.861 (0.448–1.653)	0.45	.652	77.8	.004	0.734	0.956
W vs w	4	1.419 (1.145–1.758)	3.2	.001	0	.483	0.734	0.2
Asian								
WW vs ww	3	2.857 (0.908–8.987)	1.8	.073	0	.897	0.296	0.111
WW vs Ww	3	1.427 (0.965–2.109)	1.78	.075	62.9	.068	1	0.423
WW vs Ww/ww	3	1.507 (1.033–2.199)	2.13	.033	64.3	.061	1	0.436
ww vs WW/Ww	3	0.414 (0.133–1.294)	1.52	.129	0	.853	0.296	0.056
Ww vs WW/ww	3	0.626 (0.310–1.265)	1.31	.192	59.9	.082	1	0.425
W vs w	3	1.507 (1.075–2.112)	2.38	.017	60.6	.079	1	0.438

Homozygote model, WW vs ww; heterozygous model, WW vs Ww; dominant model, WW vs Ww/ww; recessive model, ww vs WW/Ww; over-dominant model, Ww vs WW/ww; allele genetic model, W vs W.

CI = confidence interval, OR = odds ratio.

A total of 910 cases and 1411 controls who are calculated Fok-1 variant and the susceptibility to BPH in Table 5. The results indicated that null significant association between Fok-1 polymorphism and the risk of BPH (Table 5) and clinical progression of BPH in Asian. The Fok-1 polymorphism was not associated with the volume of BPH by included 2 studies. When omitted 1 study that Fok-1 variant no matching to HWE, the pooled ORs were not significant change.

**Table 5:** Results of the association between Fok-1 polymorphism and BPH risk in different ethnicities.

Comparison	Studies	Overall effect			Heterogeneity		Public bias	
		OR (95% CI)	Z-score	*P* value	*I*^2^ (%)	*P* value	Begg test	Egger test
Whole								
WW vs ww	5	0.786 (0.546–1.131)	1.3	.194	39.7	.156	0.462	0.301
WW vs Ww	5	0.918 (0.643–1.309)	0.48	.635	58.6	.047	1	0.884
WW vs Ww/ww	5	0.908 (0.449–1.219)	0.54	.591	60.8	.037	1	0.868
ww vs WW/Ww	5	1.145 (0.625–2.097)	0.44	.661	61.5	.034	0.462	0.437
Ww vs WW/ww	5	1.142 (0.814–1.602)	0.77	.444	65.4	.021	1	0.864
W vs w	5	1.057 (0.732–1.524)	0.29	.768	82.2	0	1	0.506
Caucasian								
WW vs ww	3	0.670 (0.287–1.565)	0.93	.355	0	.856	0.296	0.002
WW vs Ww	3	0.956 (0.501–1.822)	0.14	.89	53.4	.17	0.296	0.014
WW vs Ww/ww	3	1.068 (0.680–1.678)	0.29	.775	22.9	.273	0.296	0.138
ww vs WW/Ww	3	0.841 (0.367–1.928)	0.41	.682	35.8	.211	0.296	0.131
Ww vs WW/ww	3	1.158 (0.572–2.344)	0.41	.684	76.4	.014	1	0.403
W vs w	3	1.324 (0.899–1.951)	1.42	.156	53.3	.117	0.296	0.303
Asian								
WW vs ww	2	0.814 (0.544–1.219)	1	.318	83.7	.013		
WW vs Ww	2	0.75 (0.544–1.219)	0.75	.452	67.7	.07		
WW vs Ww/ww	2	0.824 (0.492–1.381)	0.73	.463	79.2	.028		
ww vs WW/Ww	2	1.551 (0.518–4.646)	0.78	.433	80.9	.022		
Ww vs WW/ww	2	1.173 (0.824–1.671)	0.89	.376	59.9	.114		
W vs w	2	0.821 (0.501–1.347)	0.78	.435	87.5	.005		

Homozygote model, WW vs ww; heterozygous model, WW vs Ww; dominant model, WW vs Ww/ww; recessive model, ww vs WW/Ww; over-dominant model, Ww vs WW/ww; allele genetic model, W vs W.

CI = confidence interval, OR = odds ratio.

### 3.2. Sensitive analysis and publication bias

The results of sensitivity analysis for VDR gene polymorphisms including Fok-1, Bsm-1, Taq-1, and Apa-1 were displayed respectively as forest maps in Supplementary 1a–d, Supplemental Digital Content, http://links.lww.com/MD/L802; http://links.lww.com/MD/L803; http://links.lww.com/MD/L804; http://links.lww.com/MD/L805. Furthermore, we implemented Begg test and Egger test to estimate the potential publication bias (Tables 2–4). Some assessments of the funnel plots indicated no publication bias (Supplementary 2a–d, Supplemental Digital Content, http://links.lww.com/MD/L806; http://links.lww.com/MD/L807; http://links.lww.com/MD/L808; http://links.lww.com/MD/L809).

## 4. Discussion

There is an increasing number of studies on the correlation between VDR polymorphisms and the risk of BPH, but the results remained inconclusive.^[23,24]^ VDR may be may be a potential target gene for the treatment of BPH.^[25,26]^ BPH was considered a hereditary disease, and the difference in VDR genotypes had a significant influence on the occurrence of BPH.^[20]^ A meta-analysis with 7 literature was implemented in 2014, which seemed to be out of date because of new appeared analysis.^[9]^ The shortage of this study was that they only carried out the overall analysis besides Taq-1 polymorphism. We could not rule out the difference in ethnicity which might influence the results. Furthermore, the results came to change essentially when omitting some studies and most of the results had high heterogeneity. Finally, we performed the newest meta-analysis to assess the relationship between VDR gene polymorphisms and the BPH risk. The detailed subgroup analyses and sensitivity analyses of all the polymorphisms are conducted. The low heterogeneity results of this meta-analysis could provide more comprehensive results to determine the association between VDR gene polymorphisms and the BPH risk.

According to the outcomes of our meta-analysis, 3 of 4 VDR polymorphisms (Fok-1, Bsm-1, Apa-1) were not associated with the BPH risk in overall populations, which were similar to the results of prior meta-analysis.^[9]^ Our meta-analysis omitted a cohort study by Mullan et al,^[27]^ which included the Taq-1 and Bsm-1 polymorphisms because its Newcastle Ottawa Scale was <5 points. The data obtained in our study suggested that the Bsm-I variant recessive model in Caucasians and over-dominant model in Asians might be protective, while contrary to the over-dominant model in Caucasians and recessive model in Asians. These results complemented the empty content of the previous meta-analysis on the Bsm-1 ethnicity subgroup due to a lack of data. The over-dominant model in Caucasians indicated a high heterogeneity concerning clinical heterogeneity, and the heterogeneity decreased when omitted El-Ezzi et al.^[16]^ Thus, we were cautious about explaining the correlation between the over-dominant model and the risk of BPH in Caucasians. We observed that the allele genetic model of Apa-1 polymorphism increased the risk of BPH in Caucasians. significant heterogeneity between trials of Fok-1 polymorphism was detected, as the distributions of genetic models and the study design were quite different. However, contrary to the low heterogeneity of the Fok-1 variant in the last meta-analysis, the experimental design and the number of included studies might play a key role in the high heterogeneity in our analysis. In addition, we found that almost studies accorded with HWE, which meant we could exclude the HWE might affect the stabilization and heterogeneity of results.

Furthermore, our result suggested that significant relationship between the Taq-1 variant and the risk of BPH exited in multi-populations. When analyzing the association between Taq-1 polymorphism and the BPH risk in multiple populations, the homozygote model, heterozygous model, dominant model, and allele genetic model might increase the risk of BPH. However, the recessive model got a declining result. We also observed that the homozygote model, heterozygous model, and dominant model in the Taq-1 variant in the Caucasian population got auxo-action for the BPH risk, while the recessive model was negative. There was low heterogeneity in the above results which meant these studies were suitable to be pooled. When in the Asian population, the result showed a significant association between the dominant model and the allele genetic model.^[28]^ The results indicated that ethnic differences could significantly alter the distribution of gene polymorphism models. However, it was worth noting that the heterogeneity of the dominant model and allele genetic model in Asians was >50%. After comparing data by software, it was considered that all the heterogeneity was derived from Habuchi et al.^[20]^ But we couldn’t explain the heterogeneity at present. So, the results come out in Asians to explain the correlation was conservative. In general, the obvious correlation between Taq-1 and BPH is different from that in the last meta-analysis, the different results might be caused by the quality and quantity of the included studies. When we considered the association between the prostate volume and Fok-1 and Taq-1 polymorphisms, null meaningful results were found. More studies should confirm this hypothesis in the future. There was no clinical application of the VDR polymorphisms to predict the risk of BPH.^[29]^ According to current analysis results, Taq-1 polymorphisms might be a “star biomarker” to predict the appearance of BPH.

It should be mentioned that There were several limitations in the current meta-analysis. First of all, only 10 eligible trials were included in our analysis, which meant we need more studies to evaluate the relationship between VDR polymorphisms and the BPH risk that could increase the statistical power. As we all know, there were more than 14 VDR polymorphisms in the VDR gene.^[30]^ So, we needed more studies to prove whether any association between other polymorphisms and the risk of BPH. Furthermore, International Prostate Symptom Score, prostate volume, and other prostatic progression indexes should be considered in the future related to VDR polymorphisms. Second, publication and language bias could be present because we only searched for publications in Chinese and English. In addition, because of the lack of enough available information, only a sub-analysis based on ethnicity was performed. More detailed national and regional population studies could be carried out in the future. According to the mentions of Brustad et al^[31]^ and Ruiz-Ballesteros et al,^[30]^ we hypothesized that gene-gene, as well as gene-environment interactions, might interfere with our results. Some similar studies could be conducted later on to verify gene-gene and gene-environment interactions. So, we could not ignore these potential parameters and we should be able to discuss their relationship further.

## 5. Conclusion

In conclusion, this meta-analysis might be the most updated one to assess the association between VDR polymorphisms and the BPH risk. With the limited number of included publications, our analysis evaluated the association of VDR polymorphisms with the risk of BPH, but not the progression of BPH. Thus, larger sample size studies should be carried out.

## Author contributions

Conceptualization, data curation, formal analysis, funding acquisition, investigation, methodology, project administration, resources, writing—original draft, writing—review & editing: Li Ruan.

## Supplementary Material
















