# Supplementary material for: Association between vitamin D receptor gene polymorphisms and genetic susceptibility to benign prostatic hyperplasia: A systematic review and meta-analysis
Source: Medicine (Baltimore). 2024 Mar 1;103(9):e37361. doi: 10.1097/MD.0000000000037361 (PMC10906597; doi:10.1097/MD.0000000000037361)
Supplement: Supplementary file 3 [file medi-103-e37361-s003.doc]

The results of sensitivity analysis for VDR gene polymorphisms Taq-Ⅰwere displayed respectively as forest maps.


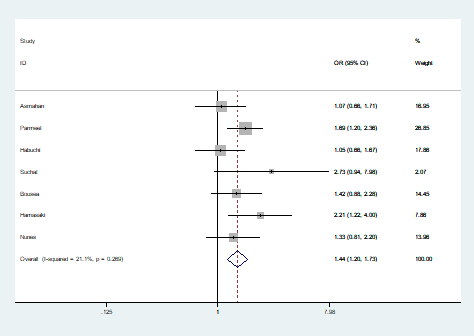


Taq-Ⅰ T VS t forest map
