# Supplementary figures and images for: Association between vitamin D receptor gene polymorphisms and genetic susceptibility to benign prostatic hyperplasia: A systematic review and meta-analysis
Source: Medicine (Baltimore). 2024 Mar 1;103(9):e37361. doi: 10.1097/MD.0000000000037361 (PMC10906597; doi:10.1097/MD.0000000000037361)

Some assessments of the funnel plots indicated no publication bias.


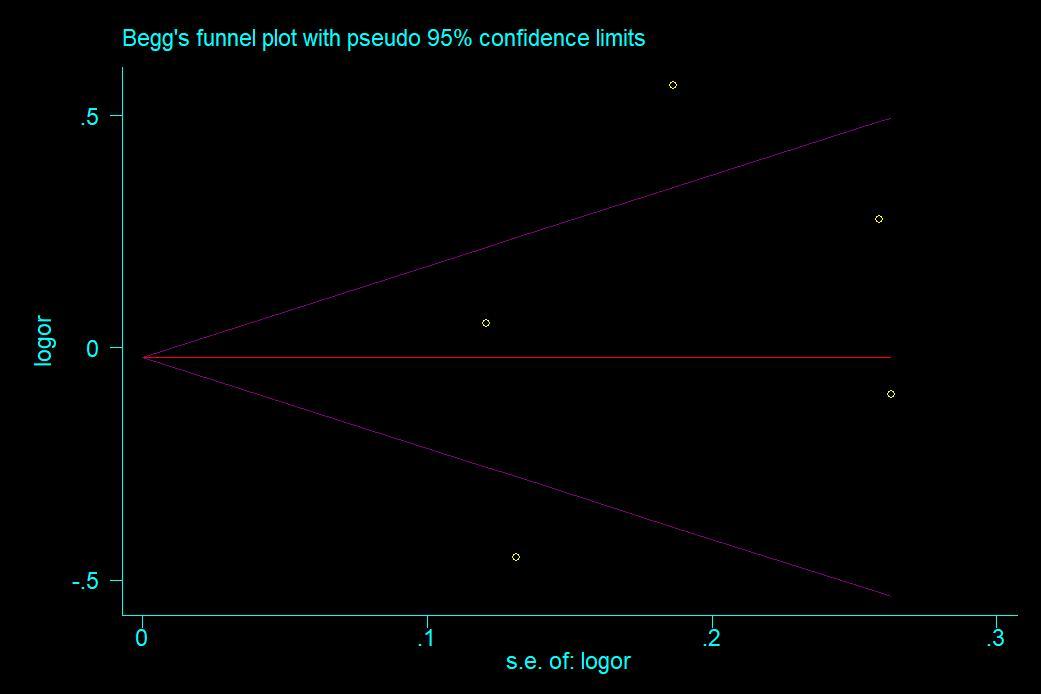


Fok-ⅠF VS f

Supplement: Supplementary file 5 [file medi-103-e37361-s005.doc]

Some assessments of the funnel plots indicated no publication bias.


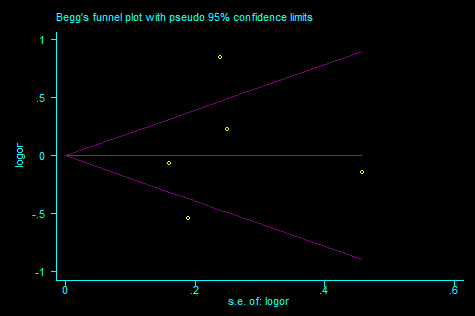


Bsm-ⅠB VS b

Supplement: Supplementary file 6 [file medi-103-e37361-s006.doc]

Some assessments of the funnel plots indicated no publication bias.


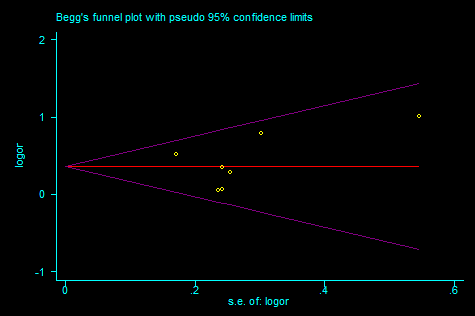


Taq-ⅠT VS t

Supplement: Supplementary file 7 [file medi-103-e37361-s007.doc]

Some assessments of the funnel plots indicated no publication bias.


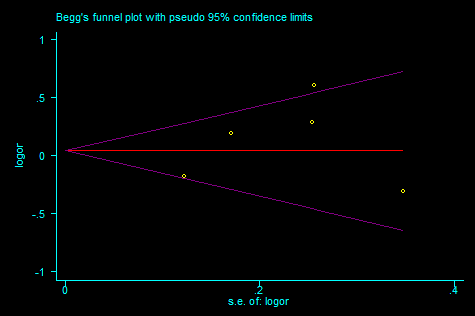


Apa-ⅠA VS a

Supplement: Supplementary file 8 [file medi-103-e37361-s008.doc]
